# Investigation of Pitch and Tone Preference of Preschool Children in Mandarin

**DOI:** 10.3390/bs16030460

**Published:** 2026-03-20

**Authors:** Minmin Yin, Surina Zhang, Hongyun Zhu, Jieyi Huang, Shengnan Ge, Baoming Li

**Affiliations:** 1 Zhejiang Philosophy and Social Science Laboratory for Research in Early Development and Childcare, Hangzhou Normal University, Hangzhou 311121, China; yinminmin@hznu.edu.cn (M.Y.); 2021112004178@stu.hznu.edu.cn (S.Z.); 2023112004224@stu.hznu.edu.cn (J.H.); 2 Department of Special Education, Faculty of Education, Hangzhou Normal University, Hangzhou 311121, China; 3 Institute of Brain Science, Hangzhou Normal University, Hangzhou 311121, China; 4 Department of Physiology, School of Basic Medical Sciences, Hangzhou Normal University, Hangzhou 311121, China; 5 Hangzhou Normal University Affiliated Sci- and Tech-City Kindergarten, Hangzhou 311121, China; 2024112004143@stu.hznu.edu.cn; 6 Department of Education and Rehabilitation, Faculty of Education, East China Normal University, Shanghai 200062, China; 52211300019@stu.ecnu.edu.cn

**Keywords:** pitch preference, tone preference, preschool children, Mandarin

## Abstract

Child-directed speech (CDS) is characterized by a suite of exaggerated acoustic features, with elevated fundamental frequency (pitch) being a prominent and widely adopted component. While caregivers and educators frequently use high-pitch speech with young children, its perceptual preference among preschool-aged children, particularly in tonal languages like Mandarin, remains empirically unclear. This study aimed to investigate Mandarin-speaking preschoolers’ explicit preferences for manipulated pitch levels at the sentence frame while also examining the potential influence of lexical tone. Ninety-four children aged 3–6 years completed a binary forced-choice preference task. They listened to sentences systematically varying in three pitch levels (high, normal, low F_0_) and five tone conditions (the four Mandarin lexical tones and a mixed-tone condition), with other acoustic parameters controlled. Results revealed that children showed no significant preference for high-pitch over normal-pitch speech. However, they exhibited a strong aversion to low-pitch speech. Furthermore, children’s pitch-level preferences were not modulated by the lexical tone of the sentences. These findings clarify that Mandarin-speaking preschoolers do not inherently prefer the high pitch typical of CDS over a normal speaking voice but are distinctly unfavorable toward low pitch. The study suggests that effective, listener-centered communication in early childhood settings may prioritize avoiding unusually low pitch rather than deliberately raising pitch, offering evidence-based guidance for pedagogical practice and adult–child interaction.

## 1. Introduction

Speech is a fundamental medium for human communication, enabling a listener to accurately perceive a speaker’s intended message. Beyond its role as an acoustic signal, speech operates as a complex system in which nuanced meanings are encoded and transmitted through the vocal apparatus (Xu, 2019). Among the various acoustic cues that structure speech, pitch—the listener’s perceptual correlate of fundamental frequency—plays a particularly critical role. It not only serves as a key attribute of the speech signal but also carries substantial communicative weight, such as conveying emotional states (Gussenhoven, 2004). Physically, pitch is primarily determined by the frequency of vocal fold vibration, though it can also be influenced by factors like sound pressure and duration (Zheng & Brette, 2017). Given its dual role as both a structural and functional element of speech, understanding how pitch is processed is essential for a complete picture of speech perception. While extensive research has examined pitch processing in adults, far less is known about the developmental trajectory of this ability in early childhood. The present study therefore aimed to investigate how young children perceive and process pitch variations in speech.

### 1.1. Speech of Preschool Teachers

Speaking is an auditory art. Teachers, especially preschool teachers who rely heavily on oral communication, exert a considerable influence on their special audience—students. Research on teachers’ speech suggests that students perceive teachers’ vocal qualities, including mood, pitch, and clarity, as impactful on their learning experience (Li, 2007). Acoustic analyses have further revealed that teachers modulate their speech across different contexts: for instance, elementary school teachers exhibit higher mean fundamental frequency (F_0_) and F_0_ standard deviation (F_0_SD) during instructional lectures compared to when they are simply reading aloud (Södersten et al., 2002; Subramaniam & Ramamurthy, 2020; Szabo Portela et al., 2013).

Extending this line of inquiry to early childhood education, studies have found that preschool teachers tend to exhibit slightly higher acoustic parameters than elementary school teachers. The literature offers two conceptually distinct explanations for this phenomenon. The first is an environmental adaptation account, which posits that preschool teachers raise their pitch in response to a noisier auditory environment. Because preschool children generally engage in more spontaneous and vocally active communication than older children, the elevated ambient noise—particularly the high-pitched background sound of children’s voices—may increase the speaker’s Sound Pressure Level (SPL) through the Lombard effect, consequently elevating the teacher’s pitch and resulting in higher vocal cycle and distance doses (Remacle et al., 2014). The second is a social accommodation account, which emphasizes communicative alignment. According to this view, females in particular tend to unconsciously adjust their pitch upward to match the higher fundamental frequency of children’s voices during social interactions, a form of phonetic convergence that facilitates rapport and effective communication (Szabo Portela et al., 2013). Taken together, these accounts suggest that preschool teachers’ use of high-pitched speech is contextually driven, whether by acoustic–environmental demands or by social-communicative feedback to the child.

The occupational cost of this vocal adjustment is well-documented. Acoustic analyses of female elementary school teachers’ voices, for instance, have revealed a positive association between higher pitch and self-reported throat fatigue (Moreno et al., 2022). This is likely because elevated pitch entails a higher mechanical load on the vocal folds, increasing both the frequency of vocal fold vibration and internal laryngeal constriction, which over time may contribute to vocal fatigue symptoms (Mohseni & Sandoughdar, 2016). However, while the production side of this phenomenon—teachers’ tendency to raise their pitch—has received considerable research attention, the perception side remains largely unexplored. That is, it is not yet known whether children actually perceive these pitch modifications nor whether such speech patterns are functionally effective or socially accepted by their intended young audience. This gap motivates the present study’s focus on children’s perception of pitch variations in teachers’ speech.

### 1.2. Pitch of Child-Directed Speech

In everyday life, it is common that parents and teachers speak with preschoolers in a different rhythm. Based on this reality, some experts have conducted studies on this topic, mostly on child-directed speech (CDS) or infant-directed speech (IDS) but not directly on children’s pitch preference. CDS refers to the way in which primary caregivers interact with infants and toddlers and is characterized acoustically by exaggerated pitch and pitch range, variable rhythm, slowed rate, and hyperarticulation of vowels (Genovese et al., 2020; Ratner, 2013; Spinelli et al., 2017). This suggests that pitch has a strong influence on children’s speech perception.

The literature on CDS has proposed two primary hypotheses regarding its function. The hyperarticulation hypothesis posits that caregivers enhance the acoustic contrast of phonemic boundaries in CDS to facilitate children’s speech and language acquisition (Cychosz et al., 2021; Hoff et al., 2020; Martin et al., 2015). The pragmatic hypothesis, in contrast, emphasizes the role of CDS in engaging children’s attention and conveying emotional or affective meaning (Benders, 2013; A. Chen et al., 2017; McMurray et al., 2013). While these hypotheses differ in their proposed mechanisms, both converge on the premise that CDS serves to support child-directed communication. Among the multiple acoustic and prosodic features that characterize CDS—including slower speech rate, exaggerated intonation, and expanded pitch range—pitch modulation stands out as a particularly salient cue. Elevated and variable pitch has been shown to capture children’s attention and signal affective intent (Fernald & Kuhl, 1987; Trainor & Desjardins, 2002). However, it is important to note that pitch is but one dimension of a multidimensional signal; the effects of CDS on children’s speech perception likely arise from the coordinated interplay of pitch with other acoustic and interactional features rather than from pitch alone. Understanding how children process pitch specifically—while holding other CDS features constant—requires systematic experimental investigation, which is the focus of the present study.

Acoustic analyses have consistently demonstrated that CDS differs from adult-directed speech (ADS) along multiple dimensions, including higher mean fundamental frequency (F_0_), slower speech rate, and greater F_0_ variation (Song et al., 2010). Behavioral studies further indicate that young children are sensitive to these acoustic features: two-year-olds, for instance, show significantly greater head-turning toward CDS passages characterized by higher average F_0_ and expanded F_0_ range, suggesting a continued preference for the prosodic properties of infant- and child-directed speech (Estes & Hurley, 2013; Segal & Newman, 2015). These pitch-related characteristics contribute to a distinctive rhythmic structure that captures children’s attention and has been associated with increased neural engagement and facilitated word learning in experimental contexts.

Evidence from intervention studies further underscores the developmental significance of CDS. The Infant Behavior Program, a parenting intervention delivered to mothers of infants aged 3–6 months, aims to enhance parent–infant interaction quality by reducing disruptive behaviors and promoting prosocial behavior in infants. Post-intervention analyses revealed that mothers in the experimental group exhibited a greater F_0_ range and slower speech rate compared to controls—vocal modifications consistent with the characteristic prosodic features of CDS (Morningstar et al., 2019). Importantly, these changes in maternal vocal behavior were significantly correlated with children’s vocabulary outcomes, suggesting that the adoption of CDS-style speech within the intervention context may support lexical development. However, it is important to note that such interventions involve multifaceted changes in parenting behavior, and the observed vocabulary gains cannot be attributed solely to pitch modulation. Rather, pitch is best understood as one component of a broader communicative package that includes prosodic, lexical, and interactional features, all of which likely contribute to children’s language development. Taken together, these findings indicate that children are attuned to the acoustic properties of CDS—including, but not limited to, pitch—and that their responsiveness to such features may play a role in early language acquisition.

The developmental sensitivity of maternal pitch extends well beyond early childhood. When CDS was examined in interactions with adolescents, researchers assessed the predictive value of multiple acoustic characteristics (F_0_, F_0_ range, duration, amplitude, and brightness—a perceptual correlate of the spectral centroid that reflects timbre) for child age (late childhood and early adolescence). Results showed that maternal pitch exhibited a significant negative correlation with child age, even after controlling for all other acoustic features (Leipold et al., 2022). Given that the child age range in our sample spans a period of rapid vocal and motor development, it can be inferred that maternal pitch—as the only statistically significant acoustic predictor—may reflect caregivers’ fine-tuned adaptation to their children’s changing developmental needs.

Cross-linguistic evidence further supports the robustness of pitch modulation in CDS. When Dutch-speaking mothers taught unfamiliar words to 18- and 24-month-old children, they exhibited expanded pitch range and hyperarticulated vowel spaces—prosodic modifications consistent with CDS (Han et al., 2020). Although the study found minimal differences between the two age groups, this may reflect the relatively narrow age range examined. More broadly, the characteristic prosodic features of CDS—including elevated and variable pitch—have been documented across diverse languages, including French, Italian, German, Japanese, British English, and American English (Fernald et al., 1989), pointing to a potentially universal communicative adaptation.

Taken together, these findings establish maternal pitch as a robust and developmentally sensitive correlate of caregiver speech. However, it is important to recognize that this literature has primarily focused on the production side of the phenomenon—documenting how caregivers adjust their pitch across development—rather than on the perception side, which concerns how children actually process these pitch modifications. Establishing that pitch is a salient and reliable feature of CDS is a necessary first step, but it does not in itself explain whether or how children use this acoustic cue to support speech processing and language learning. The present study therefore moves beyond describing the features of caregiver input to investigate the perceptual consequences of pitch variation from the child’s perspective—specifically, how young children perceive and discriminate pitch in speech.

### 1.3. Tone of Mandarin

As a canonical tonal language, Mandarin utilizes pitch patterns at the syllable level to distinguish lexical meaning. It has four tones (T1, T2, T3, T4)—a feature that sets it apart from non-tonal languages such as English or Spanish, where pitch variations primarily convey intonation or emotional nuance rather than semantic contrasts. The Mandarin tones carry both acoustic information and phonemic information: acoustic information refers to the tonal properties embodied in the tones, which are expressed as changes in the height of the sound; phonemic information refers to the tones with the role of discriminating meaning like phonemic segments, making the four tones of the same syllable carry different meanings. Children’s tone perception has been a hot topic of research interest, and it is mostly discussed in terms of the special and single factors affecting tone. Studies have shown that three-year-old children can tolerate change in tone in word recognition when the tone of a word is changed or unrelated tones are pronounced (Ma & Zhou, 2019). Four-year-old children have category perception of tone pattern, and 5-year-old children can distinguish between tone categories, suggesting a critical period for the development of tone perception (F. Chen et al., 2017). Even when tones are used to perceive changing syllables, the effect of tone is limited (Zhao et al., 2016). Researchers have also found that children aged 2–4 years are in the developmental stage of tone perception and that tone alone does not influence speech perception (Yang et al., 2020). The internal distinction between the four tones is found to be accurate for two tones, Tone 1 and Tone 4, but there is still confusion about Tone 2 and Tone 3, which have similar acoustic characteristics (Ma et al., 2017; R. Shi et al., 2017; Tsao, 2017). Thus, tone is not yet an important factor in children’s perception of speech in early childhood, since children at this age have not yet acquired all tones.

In tonal languages, tones also play a pivotal role, both at the syllable level for lexical contrasts and at the speech level for pragmatic functions. Mothers who use Chinese to teach children unfamiliar words apply higher F_0_ and a greater range of F_0_ than familiar words, producing a higher average pitch to accentuate unfamiliar words (Fernald et al., 1989). This suggests that even in Mandarin Chinese, CDS is consistent with the hyperarticulation hypothesis. However, some researchers have argued the opposite, suggesting that high pitch does not improve children’s perception and recognition of words and may even be detrimental to phonological learning. This perspective aligns with the pragmatic hypothesis, which posits that the pitch properties of CDS serve attentional, affective, or social functions rather than directly facilitating linguistic encoding. For instance, Gauthier and Shi (2011) provided evidence supporting this view, demonstrating that exaggerated pitch contours in infant-directed speech may primarily engage infants’ attention rather than enhance phonetic discrimination. This leads to the conclusion that Mandarin Chinese speech is equally applicable to the two main hypotheses of CDS and that the frequency of pitch can be increased to attract children to give feedback.

In tonal languages such as Mandarin Chinese, understanding speech perception requires careful differentiation among three related but distinct constructs: pitch, lexical tone, and intonation. Pitch refers to the perceptual correlate of F_0_. Lexical tone refers to the use of pitch patterns to distinguish word meanings at the syllable level (e.g., Mandarin’s four tones: T1, T2, T3, T4). Intonation, by contrast, operates at the sentence level, where pitch contours convey pragmatic or affective meaning. Critically, these levels interact: although the canonical shape of a lexical tone remains relatively stable, its phonetic realization must accommodate the rising or falling pitch patterns of sentential intonation. In this sense, lexical tone is embedded within, and modulated by, intonation (Cao, 2002).

A growing body of research has examined how these acoustic features manifest in Mandarin CDS. Some researchers found that mothers produced speech with higher overall pitch when addressing 18- and 24-month-old children during reading tasks compared to ADS—consistent with the cross-linguistic pattern of elevated pitch in CDS (Han et al., 2018). However, studies investigating whether mothers enhance tonal contrasts in CDS have yielded mixed findings. Another study examined Mandarin tone production in maternal speech and found no significant enhancement of tonal distinctions compared to ADS (Wong, 2018), suggesting that while global pitch is elevated, the relative phonological contrasts among tones may remain stable.

Within the literature on children’s tone perception, researchers have often focused on specific tone pairs that pose differential perceptual challenges. The contrast between T1 and T4 and the contrast between T2 and T3 are commonly employed, as they represent acoustically distinct and developmentally relevant tonal contrasts (A. Chen et al., 2017; L. Liu & Kager, 2017). Evidence from H. M. Liu et al. (2009) suggests that the T1–T4 contrast may be particularly enhanced in CDS, possibly due to the greater acoustic salience of their F_0_ differences compared to the more subtle T2–T3 distinction. However, because tone realization is inevitably influenced by sentential prosody, investigating children’s tone perception requires careful control of the intonational context in which tones are embedded. Thus, while intonation remains an important factor modulating speech perception in tonal utterances, rigorous experimental designs must balance tonal contrasts across comparable syllabic and prosodic environments to isolate perceptual mechanisms.

### 1.4. Current Study

To date, research on children’s pitch perception has been centered on nurturers. Children in the early childhood stage, whose main places of activity include kindergarten, are the important communicators for preschool teachers. In Norway, Steen and Englund (2022) conducted a study on the child-directed speech (CDS) of preschool teachers, focusing on the F_0_ and RangeF_0_ of vowels in Norwegian. Their analysis of the acoustic parameters of teachers’ CDS in the context of teacher–student interaction revealed that preschool teachers, like caregivers, increase the variation in F_0_ and RangeF_0_ to attract children’s attention and facilitate their acquisition of language.

Therefore, we can see that most of the existing studies have examined the effects of high-pitch speech on children from the adults’ perspective, and few explore the pitch preference from the children’s perspective. Although infants seems to prefer higher-pitched faces (Cristia, 2013), a study in Norwegian showed that kindergarten children demonstrate no particular preference for high-pitch speech (H.-K. Kim et al., 2015). Given that Mandarin Chinese is the most widely spoken tonal language in the world, it provides a uniquely informative context for investigating how tone experience shapes children’s perception of pitch in speech. The presence of lexical tone as a phonemic contrast in Mandarin means that pitch variations carry meaning at the syllable level, making questions about pitch preference and perception particularly consequential for this population. The purpose of the present study is therefore to determine whether Mandarin-speaking preschool children exhibit any preference for specific pitch frequencies in spoken language. In order to control for the influence of context, a balance of utterance length, word meaning, and syllable tone were used to ensure that pitch was the only perceptual cue for children. Based on the literature reviewed above, the following hypotheses guided the present study: (1) Mandarin-speaking preschoolers will exhibit a systematic preference for pitch in child-directed speech, specifically favoring higher-pitched voices over normal- or lower-pitched voices. This hypothesis tests whether the attentional function of pitch observed in infants extends into the preschool years within a tonal language context. (2) The lexical tones of Mandarin do not modulate children’s pitch preference; that is, children’s preference for higher pitch will remain consistent regardless of whether the syllable carries Tone 1, Tone 2, Tone 3, or Tone 4. This hypothesis examines whether tonal status—a phonemically contrastive pitch property—interacts with or overrides the general preference for higher pitch in the speech signal. By examining these hypotheses, the study aims to clarify whether the pitch preferences documented in non-tonal languages generalize to Mandarin-learning children and whether tonal experience and developmental stage modulate such preferences.

## 2. Materials and Methods

### 2.1. Participants

A total of 94 children, 52 males and 42 females between 3.3–6.2 years old (M = 4.9 years old), from the Hangzhou Normal University Affiliated Future Sci- and Tech-City Kindergarten, were selected and involved in the present study, of whom there were 30 children aged 3–4 years, 31 aged 4–5 years, and 33 aged 5–6 years. The following inclusion criteria were applied, and eligibility was determined through a combination of caregiver report, teacher interview, and researcher-administered assessments prior to the experiment: (1) Normal hearing sensitivity, defined as a mean pure-tone hearing threshold of less than 25 dB in both ears at 500, 1000, 2000, and 4000 Hz, as confirmed by individual screening audiometry conducted in a quiet room at the kindergarten. (2) Native Mandarin Chinese speaker with typical language development. This was initially established through a caregiver-reported questionnaire regarding the child’s language background and any history of language delay. This information was corroborated by the child’s kindergarten teacher. Additionally, all children were required to demonstrate the ability to follow simple, multi-step verbal instructions during a preliminary familiarization session with the experimenter, ensuring they could comprehend the task demands. (3) No intellectual, developmental, or systemic disorders, as reported by caregivers via a health screening questionnaire. This was further verified by reviewing the child’s annual health examination records provided by the kindergarten, which included routine pediatric assessments. (4) Good physiological and psychological state on the day of testing, defined as the absence of self- or caregiver-reported symptoms of acute illness (e.g., cold, fever, cough, ear pain) and the exhibition of normal, attentive behavior during the task. Data collection was rescheduled for any child who appeared fatigued, fussy, or unwilling to participate. The experimental procedure involving the kindergarten children was reviewed and approved by the Ethics Review Committee of Hangzhou Normal University (approval number: 2023013).

### 2.2. Speech Stimuli

Most studies on pitch use self-compiled speech stimuli, and the design frequently involves creating several semantically neutral sentences and having them recorded by audio recorders. Given that the subjects in the present study were kindergarten children aged 3–6 years old and considering their language and cognitive development levels, the speech stimuli were prepared according to the following principles: (1) simple sentences with subject–verb–object focus and length of 5 words; (2) commonly used words in daily life that are simple and easy to understand; and (3) semantically neutral phrases without any specific emotional tendencies.

**Figure 1:**
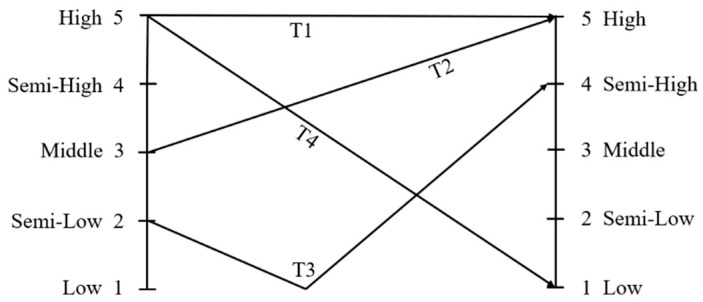
The four tones (T1–T4) in Mandarin with five-level pitches (1–5).

Mandarin Chinese exhibits four tones (T1, T2, T3, T4; see Figure 1). When preparing the speech stimuli for the experiments, we designed two sentences for each of the four tones (see Table 1). We also designed two mixed-tone sentences (Mixed-T) (see Table 1). Thus, a total of 10 sentences were used in the present study. Each of the 10 sentences were read and recorded in three frequencies: high, normal and low pitches (see Table 2).

### 2.3. Recording Procedure

The compiled speech stimuli were recorded by an adult female native speaker of Mandarin Chinese who holds a certificate of Putonghua Proficiency Level 2A (indicating a high level of fluency and accuracy in pronunciation, as graded by the National Language and Writing Work Committee of China). The data issued by the Ministry of Education of China in 2021 showed that there were a total of 2,848,600 female full-time kindergarten teachers in the whole country, accounting for 97.78% of all full-time kindergarten teachers nationwide. Hence, the recorded speech stimuli spoken by a female are more in line with the preschool environment in China. Recordings were made in a sound-insulated room with an ambient noise below 30 dB using Sound Forge 9.0 (Magix Software GmbH, Berlin, Germany) with the following software parameters: single-channel, 16-bit and sampling frequency of 44,100 Hz. Each sentence was recorded with high, normal and low pitches. The recordings were processed using the professional audio processing software Adobe Audition CS6 (Adobe Inc., San Jose, CA, USA), and noise reduction was performed on each recording. All the recorded sentences were stored in the same folder.

Thus, the 10 test sentences were recorded with high, normal and low pitch, respectively, producing a total of 30 sentences. The 30 test sentences were subjectively listened to and judged by 8 graduate students who were well trained to judge speech stimuli, and the results were fed back to the experimenter. The sentences with the highest pitch accuracy rate were selected for inclusion in the formal testing experiments.

### 2.4. Acoustic Analysis

To ensure that the speech stimuli conformed to the typical characteristics of high, normal and low pitch, they were analyzed for acoustic parameters with a Dr. Speech (DRS) device (Tiger Electronics, Inc., Vernon Hills, IL, USA) (H. Kim et al., 2019) to derive the F_0_, F_0_SD, RangeF_0_, rate, and intensity for each sentence (Table 2). F_0_ and intensity values were extracted from the midpoint of each sentence to obtain stable and representative measurements, minimizing coarticulatory effects from syllable boundaries. Also, syllable duration did not differ significantly. Moreover, to verify that the high-, normal-, and low-pitch stimuli differed significantly in the intended acoustic dimensions while being controlled in others, we first conducted a series of one-way repeated-measures Analyses of Variance (ANOVAs), with pitch (high, normal, low) as the within-subject factor, separately for each acoustic parameter: mean F_0_, F_0_ standard deviation (F_0_SD), speaking rate, and mean intensity. Where the main effect of pitch was significant (*p* < 0.05), we proceeded with post hoc paired-sample *t*-tests (with Bonferroni’s correction for multiple comparisons) to examine differences between each pair of pitch conditions. The results of these pairwise comparisons are presented in Table 3.

According to the average Chinese speech fundamental frequency norm, the mean value of adult female fundamental frequency is 230 Hz, the range of standard deviation of one fundamental frequency is ±24, and the range of standard deviation of two fundamental frequencies is ±48 (Huang et al., 2017). Therefore, the speech fundamental frequencies of all the three pitches of the recorders in the present study met the requirements of the norm, with no voice quality problems, and the frequency ranges of the three pitches of the recordings were even slightly wider than the deviation range of the fundamental frequencies of the norm.

Statistical analysis of the acoustic parameters of the speech stimuli showed that the differences in F_0_ and F_0_SD were highly significant between the normal, high and low pitches (Table 3), indicating that these parameters were effectively manipulated by our pitch-level change. Previous longitudinal studies of other languages (Sri Lankan Tamil, Tagalog, and Korean) revealed that the rate of CDS is significantly slower than that of ADS (Ko, 2012; Narayan & McDermott, 2016), suggesting that rate is an important feature in CDS and needs to be controlled in studying intonation. Therefore, to ensure that the pitch variations were not confounded by other acoustic parameters, we additionally analyzed the speech rate (measured in syllables per second) and mean amplitude (measured in decibels, dB) of the recorded stimuli. One-way ANOVAs revealed no significant differences among the high-, normal-, and low-pitch conditions in terms of either speech rate (*p* > 0.05) or mean amplitude (*p* > 0.05). These null results confirm that the three pitch conditions were acoustically comparable on these dimensions, supporting the validity of the pitch manipulation as the primary distinguishing feature of the stimuli.

### 2.5. Perceptual Experiment

After the determination of the final 30 sentences as speech stimuli, they were edited into the E-prime program with the E-prime 3.0 software (Psychology Software Tools, Inc., Pittsburgh, PA, USA). The program consisted of two blocks: practice and experiment blocks. Due to the young age of the participants and their limited text comprehension, the experimental instructional phrases were explained to them verbally by the experimenter during the practice block. The practice block contained three fixed sentences, namely, the sentence “树上有小鸟 (shù shàng yǒu xiǎo niǎo)”, which means “The tree has birds”, with normal, low and high pitches respectively. After listening to each sentence, participants were asked to make their preference for each of the three pitches (“like” or “dislike”). After participants understood the procedure, the experimenter pressed a key to enter the experiment block.

**Figure 2:**
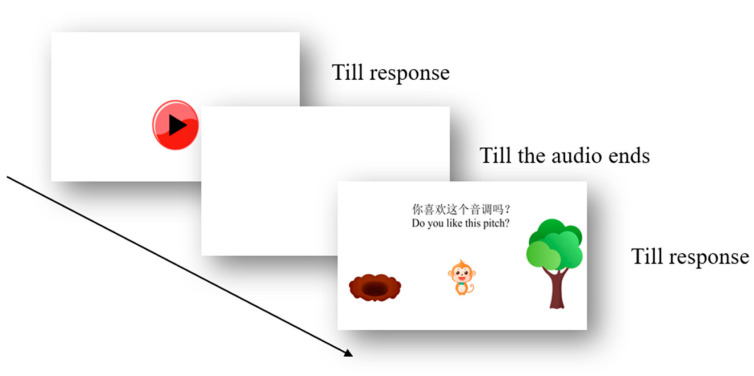
E-prime procedure for a trial of the task.

Taking into account the level of language cognitive development of children aged 3–6 years, the response options in the experimental block were set in the form of a mini-game with cartoon pictures. Thirty sentences were presented randomly. Each trial began after pressing the audio button on the touch screen, and then a sentence would appear. After the audio was presented, a cartoon “monkey” appeared in the central screen, a “tree” on the right screen and a “pit” on the left screen. Participant expressed “like the pitch” by dragging the monkey onto the tree, and “dislike the pitch” by dragging the monkey into the pit. Figure 2 shows the experimental procedure of a trial. For data coding and analysis, each individual trial yielded a binary response. A ‘like’ response (dragging the monkey to the tree) was coded as 1, and a ‘dislike’ response (dragging the monkey to the pit) was coded as 0.

### 2.6. Procedure

Before the formal experiment started, six children (one male and one female in each age group) were selected for pre-experiment. Each participant completed 30 trials in total, corresponding to the 10 unique sentences (see Table 1) each presented at three pitch levels (high, normal, low). Therefore, for each participant, each unique combination of the within-subject factors—pitch (3 levels) and tone (5 levels: T1, T2, T3, T4, Mixed-T)—was represented by 2 trials (2 sentences per tone condition). The results showed that the children could understand the experimental rule and distinguish among the three different pitches used in the present study.

The formal experiments were performed in a quiet room with background noise below 30 dB. Each participant was brought into the testing room and given brief guidance for task performance. In order to ensure the participant understood the experimental rule, he or she was presented with the sentence “树上有小鸟 (shù shàng yǒu xiǎo niǎo)” with normal, low and high pitches respectively for preference selection, as in the practice block.

In the experimental block, a trial started by displaying a start button on the touch screen. The participant was instructed with the following: “little friend, please press this start button, and you will hear a phrase. Please have a try.” Upon pressing the start button, the screen changed to blank, and an auditory sentence was played. Thereafter, the choice pictures were presented on the screen, with a cartoon monkey at the central screen and a tree and a pit on its right and left respectively. The participant was instructed at this moment “you just heard a phrase. If you like the pitch of this sentence, drag the monkey onto the tree, and if you dislike the pitch of the sentence, drag the monkey to the pit. Do you like this pitch?” A trial was completed after the participant made a selection between “like” and “dislike” by moving the cartoon monkey. Each participant was required to perform 30 trials and took approximately 8 min to complete all of the task trials.

### 2.7. Data Analysis

The present study used a 3 × 3 × 5 three-factor mixed design to investigate pitch preference in a total of 94 Mandarin preschool children. The dependent variable was pitch preference (“like” or “dislike”). The between-subject independent variable was age (3–4, 4–5 and 5–6 years). The first within-subject independent variable was pitch at the three levels (low, normal and high), and the second one was tone at the five levels (T1, T2, T3, T4 and Mixed-T).

During the experiment, both children’s reaction times and looking times were recorded for each trial. It is important to note that these measures were not treated as dependent variables in the main analysis. Rather, they were used to compute the primary dependent variable—the “like/dislike response”—which reflects children’s pitch-level preferences. Specifically, reaction time provided an index of processing speed, while looking time served as a measure of attention and preference. The operationalization of the like/dislike response from these measures is described in detail in the Results section.

After all behavioral tests were completed, the data were exported from the E-prime program. Data processing and statistical analysis of pitch preference were conducted by using the SPSS 26.0 statistical software (IBM Corporation, Armonk, NY, USA).

## 3. Results

Table 4 shows the descriptive statistics of the pitch preference rankings of the participants. The dependent variable for analysis was the average ‘like’ score for each condition. For each participant and for each specific pitch × tone condition, the scores (0 or 1) from the two corresponding trials were summed and then averaged, resulting in a mean score ranging from 0 to 1. The mean values and standard deviations reported in Table 4 and the statistical analysis are based on these averaged scores across the 94 participants. As shown, all of the three groups of children had the lowest preference ranking for low pitch at all of the tones. The 3–4-year-old children demonstrated the highest preference for high pitch at T2 and T3 and for normal pitch at T1, T4 and Mixed-T. The 4–5-year-old children exhibited the highest preference for high pitch at T1, T2, T3 and T4 but not at Mixed-T. The 5–6-year-old children displayed the highest preference for high pitch at all tones. Thus, all of the three groups of children showed the highest preference for high pitch at T2 and T3. Table 5 shows the results of the within-subjects variable sphericity test.

As is shown in Table 5, the within-subject variables, namely pitch and pitch × tone, did not satisfy the sphericity test hypothesis (*p* < 0.05), so the ANOVA (Greenhous e-Geisser) results were used, and the ANOVA results are shown in Table 6. The main effect of pitch was significant (F = 93.536, *p* < 0.001), and that of tone was significant (F = 2.863, *p* < 0.05). The interaction effect between pitch and age was significant (F = 15.119, *p* < 0.001), but that between tone and age and between pitch and tone was not. The three-way interaction of pitch, tone and age was also not significant. Since the main effects of pitch and tone were significant and both had two or more levels, the interaction between pitch and age was also significant. Multiple comparisons were made for both pitch and tone factors. Post hoc pairwise comparisons were conducted using paired-sample *t*-tests with Bonferroni’s correction for multiple comparisons (adjusted alpha level of 0.0167).

### 3.1. Pitch

**Figure 3:**
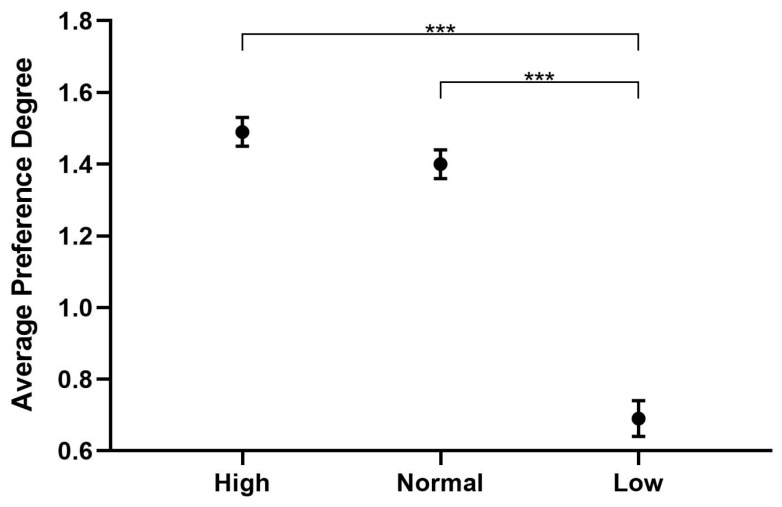
Rankings for the pitch preference of the preschool children. *** *p* < 0.001.

The results of the descriptive statistics regarding pitch were 1.49 ± 0.04, 1.40 ± 0.04, and 0.69 ± 0.05 for the preference of high, normal and low pitches, respectively (Figure 3). Inter-pitch comparison of preference ranking is shown in Table 7. As shown, the preference ranking for low pitch was extremely significantly lower than for high pitch and normal pitch (*p* < 0.001), while the preference rankings for high pitch and normal pitch were not statistically significant (*p* > 0.05).

### 3.2. Tone

**Figure 4:**
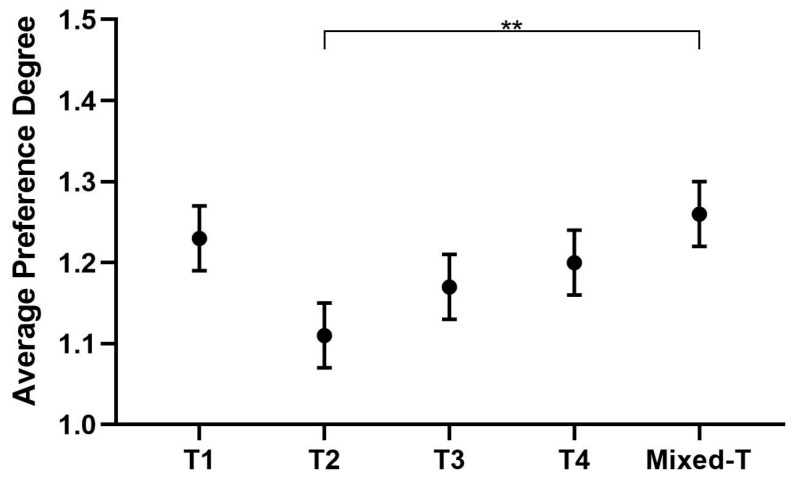
Rankings for the tone preference of the preschool children. ** *p* < 0.01.

The results of descriptive statistics regarding tones were 1.23 ± 0.04, 1.11 ± 0.04, 1.17 ± 0.04, 1.20 ± 0.04 and 1.26 ± 0.04 for the preference of T1, T2, T3, T4 and Mixed-T tones, respectively (Figure 4). Inter-tone comparison of preference ranking is shown in Table 8. As can be seen, a significant difference was only detected between T2 and Mixed-T (*p* = 0.003), suggesting that children have a similar preference for tone.

### 3.3. Pitch × Age

**Figure 5:**
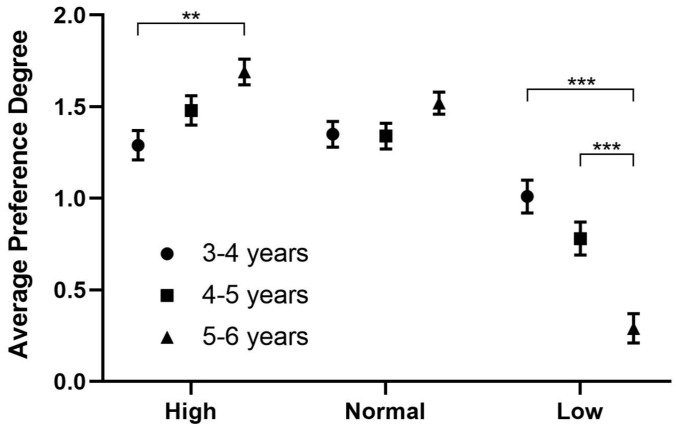
Analysis for the interaction of pitch and age. ** *p* < 0.01; *** *p* < 0.001.

Figure 5 shows the results of the simple effects test. There was no difference in the preference for normal pitch among the three age groups of preschool children. The preference of the older age group for high pitch was not significantly different from that of the middle age group (*p* = 0.239) but was significantly higher than that of the younger age group (*p* = 0.001). Interestingly, while reaction times for high and normal pitches decreased significantly with age—indicating developmental changes in processing speed—the relative preference for high and normal pitches (as indexed by looking time, from which the like/dislike response was derived) did not change significantly across age groups. However, the preference for low pitch was significantly reduced in the older age group of preschool children (*p* < 0.001).

## 4. Discussion

The present study examined the pitch preference of kindergarten children in the context of Mandarin and found that post hoc analyses following the non-significant main effect of tone revealed one specific significant difference: children showed a significantly different preference for Tone 2 compared to the mixed-tone condition (*p* = 0.032). No other pairwise comparisons among tones reached significance, but they exhibited a strong aversion to the low pitch. Moreover, the preschoolers showed no preference for tones.

### 4.1. Pitch Preference

Studies on English comprehension of 3–6 year-old children for typical subject–verb–object (SVO) inflections have revealed that children’s comprehension is enhanced by CDS as they become proficient in inflections and language morphology (Foursha-Stevenson et al., 2017). If CDS expresses speech structures that children have not yet mastered, children increase their attention to CDS, and in turn, CDS enhances children’s comprehension.

Our result is inconsistent with previous findings in CDS, where mothers are thought to subconsciously raise their pitch when talking to infants (Estes & Hurley, 2013; Morningstar et al., 2019). It is explained that high-pitched CDS captures the attention of infants and enables imitation by them (Han et al., 2020; J. Shi et al., 2022). The present study found that preschool children do not have a specific preference for high pitch versus normal pitch, but they do have a strong aversion to the low pitch. This inconsistency may be related to the age of the children. Children in early childhood are less sensitive to pitch, and previous research has shown that they cannot accurately distinguish between different pitch levels (Deroche et al., 2012). However, kindergarten children have already developed pitch sensitivity, and normal pitch is good enough to attract their attention.

Post hoc simple effects analysis of the significant pitch × age interaction revealed that the effect of age was specific to the low-pitch condition. The aversion to low pitch was significantly stronger in the 5–6-year-old group compared to the 3–4-year-old group (*p* < 0.05). In contrast, preference for high pitch and normal pitch did not differ significantly across the three age groups. At the group level, preschool children showed a similar preference for high and normal pitches, as the pairwise comparison for the main effect of pitch was not significant (*p* > 0.05). This overall similarity held despite the presence of a pitch × age interaction, which, upon analysis, was driven by a developing aversion to low pitch with age rather than by a change in preference for high pitch. It is reported that low pitch is closer to a male voice and that children are more willing to accept a female voice (Spazzapan et al., 2018). A possible explanation is that the observed age-related shift in pitch preference may reflect developmental changes in auditory processing and linguistic expectations. As children aged 5–6 gain greater proficiency in sentence comprehension, they may become more attuned to native-language phonological norms, in which lower pitch is typically associated with less salient or less engaging speech registers (e.g., adult-directed speech). Consequently, low-pitch input may be perceived as less child-appropriate or less socially engaging, leading to reduced preference. In contrast, younger children (aged 3–4), who are still in the early stages of mastering complex sentences, may rely more on raw auditory salience and thus remain more accepting of low-pitch stimuli. Thus, teachers can speak to preschool children normally, without deliberately raising pitch during teaching, but they should avoid using low-pitch speech.

### 4.2. Influence of Tone

Previous research has shown that preschool children exhibit no special preference for single or mixed tones. Singh et al. (2014) reported that children aged 18 and 24 months show sensitivity to Mandarin tone and vowel mispronunciation, either ignoring or adding tone information, suggesting that tone is not yet an important influencing factor at the vocabulary learning stage of young children. F. Chen et al. (2017) further found that the ability to recognize Mandarin tones emerges at around 6 years of age, regardless of whether tones are formally taught.

With age and knowledge, the ability for children to discriminate between tones gradually increases. The children’s pitch-level preference was largely unaffected by the lexical tone of the sentences, as evidenced by a non-significant main effect of tone. However, one isolated difference emerged, with Tone 2 eliciting a significantly different preference score compared to the mixed-tone condition. Moreover, the order of the mean F_0_ of the four tones in Mandarin is consistent and remains the same, namely, T4 > T1 > T2 > T3, as is the order of the fundamental frequency range, namely, T4 > T3 > T2 > T1 (H. M. Liu et al., 2009). This is crucial for lexical meaning at the syllable level. Thus, teachers can communicate with preschool children normally, without deliberately emphasizing a particular tone during teaching.

The present study found no interaction between pitch and tone, that is, tone did not affect pitch preference. Some researchers have pointed out that, for children, English learning must identify the specific role of pitch in marking lexical stress, while Mandarin learning must identify the role of pitch in the tone system (Quam & Swingley, 2012). Children who speak tonal languages as their mother tongue have a better ability to perceive pitch than children who speak non-tonal languages (Creel et al., 2018). In Mandarin learning, the higher overall pitch at the phrase or sentence level does not distort or obscure the lexical pitch at the syllable level, which is crucial for the retention of lexical meaning and may also provide an environmental factor for tone acquisition (Tupper et al., 2021). Furthermore, different pitch properties may serve distinct functions in the learning process: while lexical pitch variations are essential for tone discrimination and word learning, the higher phrasal pitch commonly observed in infant-directed speech may enhance attention and facilitate early language processing (H. M. Liu et al., 2009). This suggests that rather than one pitch type being universally more favorable than another, learners benefit from the complementary roles of different pitch features at multiple levels of linguistic input. Thus, regardless of the tone of the sentence, it will conform to the general pattern of intonation.

## 5. Conclusions

The present study demonstrates that, in a Mandarin-speaking context, preschool children exhibit no significant preference for high pitch relative to normal pitch, but they do show a clear dislike for low pitch. Children’s pitch-level preferences were largely independent of the specific Mandarin lexical tone, as indicated by a non-significant main effect. However, a post hoc test revealed one exception: a significant difference in preference between Tone 2 and the mixed-tone condition. These findings offer several implications for educational practice. First, given that children do not show heightened attention or preference for high-pitched speech, teachers need not feel compelled to adopt an exaggeratedly high pitch when addressing young learners—a strategy that has been associated with increased vocal fatigue. Second, and more critically, the finding that children actively dislike low-pitched speech suggests that teachers should be mindful of maintaining pitch levels that fall within a comfortable perceptual range for young listeners; excessively low pitch may reduce children’s engagement or attentional focus. Taken together, these results support a recommendation for teachers to employ a moderate, normal pitch range in classroom interactions—sufficiently high to remain perceptually accessible to children but not so elevated as to impose unnecessary vocal strain. This balanced approach aligns with both the perceptual preferences observed in children and the occupational health considerations of educators.

It should be acknowledged that this study has several limitations that warrant consideration. First, the stimulus materials were produced by only one female speaker, whose age was not specified. Given that voice frequency can vary with age—older speakers may exhibit lower pitch—it remains unclear whether the observed preference for higher pitch would generalize to voices produced by speakers of different ages or vocal characteristics. Second, the use of a single speaker limits the generalizability of the findings, as individual vocal idiosyncrasies may have influenced the results. Future research should include multiple speakers varying in age, gender, and baseline pitch to determine whether the preference for higher pitch is robust across different voice types. Additionally, extending the investigation to naturalistic speech samples and exploring whether such pitch preferences facilitate tone learning in real-world contexts would further clarify the role of caregiver pitch in early language development. Addressing these limitations will be essential for drawing more definitive conclusions about the relationship between pitch features and tone acquisition in Mandarin-learning children.

## Figures and Tables

**Table 1:** The speech stimuli used in the present study.

Tone	Speech Stimuli
T1	$\overset{gē}{哥}$ $\overset{gē}{哥}$ $\overset{chī}{吃}$ $\overset{xī}{西}$ $\overset{guā}{瓜}$ Brother eats watermelon.
	$\overset{mā}{妈}$ $\overset{mā}{妈}$ $\overset{chuān}{穿}$ $\overset{xīn}{新}$ $\overset{yī}{衣}$ Mother wears a new dress.
T2	$\overset{wáng}{王}$ $\overset{huá}{华}$ $\overset{lái}{来}$ $\overset{bá}{拔}$ $\overset{hé}{河}$ Wang Hua comes to tug-of-war.
	$\overset{yáng}{阳}$ $\overset{yáng}{阳}$ $\overset{xué}{学}$ $\overset{lán}{篮}$ $\overset{qiú}{球}$ Yangyang learns basketball.
T3	$\overset{xiǎo}{小}$ $\overset{bǎo}{宝}$ $\overset{dǎ}{打}$ $\overset{yǔ}{雨}$ $\overset{sǎn}{伞}$ Xiao Bao holds an umbrella.
	$\overset{xiǎo}{小}$ $\overset{měi}{美}$ $\overset{shǔ}{数}$ $\overset{shǒu}{手}$ $\overset{zhǐ}{指}$ Xiao Mei counts her fingers.
T4	$\overset{dà}{大}$ $\overset{xiàng}{象}$ $\overset{shì}{是}$ $\overset{dòng}{动}$ $\overset{wù}{物}$ Elephants are animals.
	$\overset{mèi}{妹}$ $\overset{mèi}{妹}$ $\overset{qù}{去}$ $\overset{xùn}{训}$ $\overset{liàn}{练}$ Sister goes to training.
Mixed-T	$\overset{xiǎo}{小}$ $\overset{huá}{华}$ $\overset{fàng}{放}$ $\overset{fēng}{风}$ $\overset{zhēng}{筝}$ Xiao Hua flies a kite.
	$\overset{gōng}{公}$ $\overset{yuán}{园}$ $\overset{yǒu}{有}$ $\overset{xiǎo}{小}$ $\overset{shù}{树}$ The park has small trees.

**Table 2:** The acoustic parameters of the speech stimuli.

Tone	Speech Stimuli	F_0_ (Hz)			F_0_SD (Hz)			RangeF_0_ (Hz)			Rate (s)			Mean Speech Amplitude (dB)		
		High	Normal	Low	High	Normal	Low	High	Normal	Low	High	Normal	Low	High	Normal	Low
T1	$\overset{gē}{哥}$ $\overset{gē}{哥}$ $\overset{chī}{吃}$ $\overset{xī}{西}$ $\overset{guā}{瓜}$	356	219	192	37	30	22	282–430	159–279	148–236	0.314	0.3	0.324	70	68	70
	$\overset{mā}{妈}$ $\overset{mā}{妈}$ $\overset{chuān}{穿}$ $\overset{xīn}{新}$ $\overset{yī}{衣}$	366	249	185	37	47	15	292–440	155–343	155–215	0.316	0.336	0.34	72	71	70
T2	$\overset{wáng}{王}$ $\overset{huá}{华}$ $\overset{lái}{来}$ $\overset{bá}{拔}$ $\overset{hé}{河}$	339	245	173	31	25	17	277–401	195–295	139–207	0.294	0.338	0.29	70	69	71
	$\overset{yáng}{阳}$ $\overset{yáng}{阳}$ $\overset{xué}{学}$ $\overset{lán}{篮}$ $\overset{qiú}{球}$	348	214	183	26	21	18	296–400	172–256	147–219	0.296	0.264	0.306	72	71	69
T3	$\overset{xiǎo}{小}$ $\overset{bǎo}{宝}$ $\overset{dǎ}{打}$ $\overset{yǔ}{雨}$ $\overset{sǎn}{伞}$	294	229	174	48	42	24	198–390	145–313	126–222	0.378	0.304	0.348	66	66	70
	$\overset{xiǎo}{小}$ $\overset{měi}{美}$ $\overset{shǔ}{数}$ $\overset{shǒu}{手}$ $\overset{zhǐ}{指}$	314	215	186	49	40	22	216–412	135–295	142–230	0.36	0.312	0.302	72	73	69
T4	$\overset{dà}{大}$ $\overset{xiàng}{象}$ $\overset{shì}{是}$ $\overset{dòng}{动}$ $\overset{wù}{物}$	347	271	166	66	56	20	215–479	159–383	126–206	0.36	0.328	0.348	72	71	71
	$\overset{mèi}{妹}$ $\overset{mèi}{妹}$ $\overset{qù}{去}$ $\overset{xùn}{训}$ $\overset{liàn}{练}$	343	215	177	53	47	24	237–449	121–309	129–225	0.302	0.27	0.356	70	69	71
Mixed-T	$\overset{xiǎo}{小}$ $\overset{huá}{华}$ $\overset{fàng}{放}$ $\overset{fēng}{风}$ $\overset{zhēng}{筝}$	336	242	184	72	67	35	192–480	108–376	114–254	0.356	0.292	0.354	66	67	70
	$\overset{gōng}{公}$ $\overset{yuán}{园}$ $\overset{yǒu}{有}$ $\overset{xiǎo}{小}$ $\overset{shù}{树}$	329	227	195	83	37	37	163–495	153–301	121–269	0.31	0.334	0.364	68	72	70

*Note: Rate(s) =* $\frac{Syllable\;duration(s)}{The\;number\;of\;syllables}$.

**Table 3:** Paired-sample statistics comparison for the acoustic parameters of the speech stimuli.

Acoustic Parameter	Paired Comparison	t	*p*
F_0_	High vs. Normal	16.16	0.00 **
	High vs. Low	25.46	0.00 **
	Normal vs. Low	6.96	0.00 **
F_0_SD	High vs. Normal	2.23	0.05 *
	High vs. Low	7.02	0.00 **
	Normal vs. Low	4.06	0.00 **
Rate	High vs. Normal	2.33	0.04 *
	High vs. Low	0.10	0.92
	Normal vs. Low	−2.40	0.04 *
Intensity	High vs. Normal	0.00	1.00
	High vs. Low	−1.01	0.33
	Normal vs. Low	−0.96	0.36

* *p* < 0.05; ** *p* < 0.01.

**Table 4:** Descriptive Statistics of Pitch Preference Rankings Among Preschool Children by Age and Tone.

Age	Tone	Pitch	Mean	SD	Age	Tone	Pitch	Mean	SD	Age	Tone	Pitch	Mean	SD
**3–4 years**	T1	High Pitch	1.33	0.76	**4–5 years**	T1	High Pitch	1.52	0.63	**5–6 years**	T1	High Pitch	1.73	0.57
		Normal Pitch	1.40	0.77			Normal Pitch	1.35	0.71			Normal Pitch	1.45	0.67
		Low Pitch	1.17	0.79			Low Pitch	0.77	0.84			Low Pitch	0.33	0.69
	T2	High Pitch	1.27	0.58		T2	High Pitch	1.45	0.72		T2	High Pitch	1.61	0.70
		Normal Pitch	1.20	0.71			Normal Pitch	1.10	0.65			Normal Pitch	1.24	0.71
		Low Pitch	1.03	0.61			Low Pitch	0.81	0.65			Low Pitch	0.24	0.50
	T3	High Pitch	1.37	0.67		T3	High Pitch	1.39	0.67		T3	High Pitch	1.70	0.59
		Normal Pitch	1.27	0.64			Normal Pitch	1.23	0.67			Normal Pitch	1.64	0.60
		Low Pitch	0.83	0.79			Low Pitch	0.84	0.86			Low Pitch	0.27	0.63
	T4	High Pitch	1.17	0.75		T4	High Pitch	1.58	0.56		T4	High Pitch	1.64	0.65
		Normal Pitch	1.40	0.72			Normal Pitch	1.52	0.63			Normal Pitch	1.58	0.61
		Low Pitch	0.87	0.68			Low Pitch	0.77	0.88			Low Pitch	0.24	0.56
	Mixed-T	High Pitch	1.33	0.71		Mixed-T	High Pitch	1.45	0.77		Mixed-T	High Pitch	1.76	0.56
		Normal Pitch	1.50	0.63			Normal Pitch	1.48	0.57			Normal Pitch	1.67	0.60
		Low Pitch	1.13	0.68			Low Pitch	0.71	0.69			Low Pitch	0.33	0.65

**Table 5:** Results for sphericity test hypothesis.

	Greenhous e-Geisser	Approx Chi-Square	df	*p*
Pitch	0.755	35.392	2	0.000
Tone	0.959	7.742	9	0.560
Pitch × Tone	0.879	51.320	35	0.037

**Table 6:** Results for three-way ANOVA.

Variable	df	F	*p*
Pitch	1.509	93.536	0.000 ***
Pitch × Age	3.019	15.119	0.000 ***
Tone	3.837	2.863	0.025 *
Tone × Age	7.674	1.013	0.425
Pitch × Tone	7.029	1.851	0.075
Pitch × Tone × Age	14.058	0.490	0.939

* *p* < 0.05; *** *p* < 0.001.

**Table 7:** Multiple comparison of the pitch preference of the preschool children.

Pitch	Pitch	*p*
High	Normal	0.051
	Low	0.000 ***
Normal	High	0.051
	Low	0.000 ***
Low	High	0.000 ***
	Normal	0.000 ***

*** *p* < 0.001.

**Table 8:** Multiple comparison of the tone preference of the preschool children.

Tone	Tone	*p*
T1	T2	0.021
	T3	0.202
	T4	0.518
	Mixed-T	0.530
T2	T3	0.190
	T4	0.102
	Mixed-T	0.003 **
T3	T4	0.582
	Mixed-T	0.051

** *p* < 0.01.

## Data Availability

The data that support the findings of this study are available on request from the corresponding author, Baoming Li (bmli@hznu.edu.cn). The data are not publicly available due to privacy and ethical restrictions.

## Abbreviations

The following abbreviations are used in this manuscript:

CDS	Child-directed speech
IDS	Infant-directed speech
ADS	Adult-directed speech

## References

[B1-behavsci-16-00460] Benders T. (2013). Mommy is only happy! Dutch mothers’ realisation of speech sounds in infant-directed speech expresses emotion, not didactic intent. Infant Behavior and Development.

[B2-behavsci-16-00460] Cao J. F. (2002). The relationship between tone and intonation in Mandarin Chinese. Zhongguo Yuwen.

[B3-behavsci-16-00460] Chen A., Stevens C. J., Kager R. (2017). Pitch perception in the first year of life, a comparison of lexical tones and musical pitch. Frontiers in Psychology.

[B4-behavsci-16-00460] Chen F., Peng G., Yan N., Wang L. (2017). The development of categorical perception of Mandarin tones in four- to seven-year-old children. Journal of Child Language.

[B5-behavsci-16-00460] Creel S. C., Weng M., Fu G., Heyman G. D., Lee K. (2018). Speaking a tone language enhances musical pitch perception in 3–5-year-olds. Developmental Science.

[B6-behavsci-16-00460] Cristia A. (2013). Input to language: The phonetics and perception of infant-directed speech. Language and Linguistics Compass.

[B7-behavsci-16-00460] Cychosz M., Edwards J. R., Bernstein Ratner N., Torrington Eaton C., Newman R. S. (2021). Acoustic-lexical characteristics of child-directed speech between 7 and 24 months and their impact on toddlers’ phonological processing. Frontiers in Psychology.

[B8-behavsci-16-00460] Deroche M. L. D., Zion D. J., Schurman J. R., Chatterjee M. (2012). Sensitivity of school-aged children to pitch-related cues. The Journal of the Acoustical Society of America.

[B9-behavsci-16-00460] Estes K. G., Hurley K. (2013). Infant-directed prosody helps infants map sounds to meanings. Infancy.

[B10-behavsci-16-00460] Fernald A., Kuhl P. K. (1987). Acoustic determinants of infant preference for motherese speech. Infant Behavior & Development.

[B11-behavsci-16-00460] Fernald A., Taeschner T., Dunn J., Papousek M., de Boysson-Bardies B., Fukui I. (1989). A cross-language study of prosodic modifications in mothers’ and fathers’ speech to preverbal infants. Journal of Child Language.

[B12-behavsci-16-00460] Foursha-Stevenson C., Schembri T., Nicoladis E., Eriksen C. (2017). The influence of child-directed speech on word learning and comprehension. Journal of Psycholinguistic Research.

[B13-behavsci-16-00460] Gauthier B., Shi R. (2011). A connectionist study on the role of pitch in infant-directed speech. The Journal of the Acoustical Society of America.

[B14-behavsci-16-00460] Genovese G., Spinelli M., Romero Lauro L. J., Aureli T., Castelletti G., Fasolo M. (2020). Infant-directed speech as a simplified but not simple register: A longitudinal study of lexical and syntactic features. Journal of Child Language.

[B15-behavsci-16-00460] Gussenhoven C. (2004). The phonology of tone and intonation.

[B16-behavsci-16-00460] Han M., De Jong N. H., Kager R. (2018). Lexical tones in Mandarin Chinese infant-directed speech: Age-related changes in the second year of life. Frontiers in Psychology.

[B17-behavsci-16-00460] Han M., De Jong N. H., Kager R. (2020). Pitch properties of infant-directed speech specific to word-learning contexts: A cross-linguistic investigation of Mandarin Chinese and Dutch. Journal of Child Language.

[B18-behavsci-16-00460] Hoff E., Core C., Shanks K. F. (2020). The quality of child-directed speech depends on the speaker’s language proficiency. Journal of Child Language.

[B19-behavsci-16-00460] Huang Z. M., Zhu Q. Y., Lu H. Y. (2017). Speech therapy.

[B20-behavsci-16-00460] Kim H., Gao S., Yi B., Shi R., Wan Q., Huang Z. (2019). Validation of the dysphonia severity index in the Dr. Speech Program. Journal of Voice.

[B21-behavsci-16-00460] Kim H.-K., Yu X. M., Lu L. J., Liu X. M. (2015). A research of tone preference of children aged 3 to 6. Studies in Early Childhood Education.

[B22-behavsci-16-00460] Ko E. S. (2012). Nonlinear development of speaking rate in child-directed speech. Lingua.

[B23-behavsci-16-00460] Leipold S., Abrams D. A., Menon V. (2022). Mothers adapt their voice during children’s adolescent development. Scientific Reports.

[B24-behavsci-16-00460] Li X. L. (2007). The investigation of the influence of teachers’ speech characteristics on students’ emotions in classroom teaching. Education Science Forum.

[B25-behavsci-16-00460] Liu H. M., Tsao F. M., Kuhl P. K. (2009). Age-related changes in acoustic modifications of Mandarin maternal speech to preverbal infants and five-year-old children: A longitudinal study. Journal of Child Language.

[B26-behavsci-16-00460] Liu L., Kager R. (2017). Perception of tones by bilingual infants learning non-tone languages. Bilingualism: Language and Cognition.

[B27-behavsci-16-00460] Ma W., Zhou P. (2019). Three-year-old tone language learners are tolerant of tone mispronunciations spoken with familiar and novel tones. Cogent Psychology.

[B28-behavsci-16-00460] Ma W., Zhou P., Singh L., Gao L. (2017). Spoken word recognition in young tone language learners: Age-dependent effects of segmental and suprasegmental variation. Cognition.

[B29-behavsci-16-00460] Martin A., Schatz T., Versteegh M., Miyazawa K., Mazuka R., Dupoux E., Cristia A. (2015). Mothers speak less clearly to infants than to adults: A comprehensive test of the hyperarticulation hypothesis. Psychological Science.

[B30-behavsci-16-00460] McMurray B., Kovack-Lesh K. A., Goodwin D., McEchron W. (2013). Infant directed speech and the development of speech perception: Enhancing development or an unintended consequence?. Cognition.

[B31-behavsci-16-00460] Mohseni R., Sandoughdar N. (2016). Survey of voice acoustic parameters in Iranian female teachers. Journal of Voice.

[B32-behavsci-16-00460] Moreno M., Calvache C., Cantor-Cutiva L. C. (2022). Systematic review of literature on prevalence of vocal fatigue among teachers. Journal of Voice.

[B33-behavsci-16-00460] Morningstar M., Garcia D., Dirks M. A., Bagner D. M. (2019). Changes in parental prosody mediate effect of parent-training intervention on infant language production. Journal of Consulting and Clinical Psychology.

[B34-behavsci-16-00460] Narayan C. R., McDermott L. C. (2016). Speech rate and pitch characteristics of infant-directed speech: Longitudinal and cross-linguistic observations. The Journal of the Acoustical Society of America.

[B35-behavsci-16-00460] Quam C., Swingley D. (2012). Development in children’s interpretation of pitch cues to emotions. Child Development.

[B36-behavsci-16-00460] Ratner N. B. (2013). Why talk with children matters: Clinical implications of infant- and child-directed speech research. Seminars in Speech and Language.

[B37-behavsci-16-00460] Remacle A., Morsomme D., Finck C. (2014). Comparison of vocal loading parameters in kindergarten and elementary school teachers. Journal of Speech, Language, and Hearing Research.

[B38-behavsci-16-00460] Segal J., Newman R. S. (2015). Infant preferences for structural and prosodic properties of infant-directed speech in the second year of life. Infancy.

[B39-behavsci-16-00460] Shi J., Gu Y., Vigliocco G. (2022). Prosodic modulations in child-directed language and their impact on word learning. Developmental Science.

[B40-behavsci-16-00460] Shi R., Gao J., Achim A., Li A. (2017). Perception and representation of lexical tones in native Mandarin-learning infants and toddlers. Frontiers in Psychology.

[B41-behavsci-16-00460] Singh L., Hui T. J., Chan C., Golinkoff R. M. (2014). Influences of vowel and tone variation on emergent word knowledge: A cross-linguistic investigation. Developmental Science.

[B42-behavsci-16-00460] Song J. Y., Demuth K., Morgan J. (2010). Effects of the acoustic properties of infant-directed speech on infant word recognition. The Journal of the Acoustical Society of America.

[B43-behavsci-16-00460] Södersten M., Granqvist S., Hammarberg B., Szabo A. (2002). Vocal behavior and vocal loading factors for preschool teachers at work studied with binaural DAT recordings. Journal of Voice.

[B44-behavsci-16-00460] Spazzapan E. A., Cardoso V. M., Fabron E. M. G., Berti L. C., Brasolotto A. G., Marino V. C. C. (2018). Acoustic characteristics of healthy voices of adults: From young to middle age. Codas.

[B45-behavsci-16-00460] Spinelli M., Fasolo M., Mesman J. (2017). Does prosody make the difference? A meta-analysis on relations between prosodic aspects of infant-directed speech and infant outcomes. Developmental Review.

[B46-behavsci-16-00460] Steen V. B., Englund N. (2022). Child-directed speech in a Norwegian kindergarten setting. Scandinavian Journal of Educational Research.

[B47-behavsci-16-00460] Subramaniam N., Ramamurthy K. (2020). Effect of mode of delivery and background noise on speech characteristics of talkers in a classroom environment. Building Acoustics.

[B48-behavsci-16-00460] Szabo Portela A., Hammarberg B., Södersten M. (2013). Speaking fundamental frequency and phonation time during work and leisure time in vocally healthy preschool teachers measured with a voice accumulator. Folia Phoniatrica et Logopaedica.

[B49-behavsci-16-00460] Trainor L. J., Desjardins R. N. (2002). Pitch characteristics of infant-directed speech affect infants’ ability to discriminate vowels. Psychonomic Bulletin & Review.

[B50-behavsci-16-00460] Tsao F. M. (2017). Perceptual improvement of lexical tones in infants: Effects of tone language experience. Frontiers in Psychology.

[B51-behavsci-16-00460] Tupper P., Leung K. W., Wang Y., Jongman A., Sereno J. A. (2021). The contrast between clear and plain speaking style for Mandarin tones. The Journal of the Acoustical Society of America.

[B52-behavsci-16-00460] Wong P. (2018). Mothers do not enhance tonal contrasts in child-directed speech: Perceptual and acoustic evidence from child-directed Mandarin lexical tones. The Journal of the Acoustical Society of America.

[B53-behavsci-16-00460] Xu Y., Katz W. F., Assmann P. F. (2019). Prosody, tone and intonation. The routledge handbook of phonetics.

[B54-behavsci-16-00460] Yang W. Q., Xiao R., Liang D. D. (2020). Lexical tone perception mechamism in 2- to 4-year-old Mandarin-speaking children in the pre-attention stage. Acta Psychologica Sinica.

[B55-behavsci-16-00460] Zhao R., Wang X. J., Yang J. F. (2016). The role of tone in Chinese syllable perception. Acta Psychologica Sinica.

[B56-behavsci-16-00460] Zheng Y., Brette R. (2017). On the relation between pitch and level. Hearing Research.

